# Switchable deep eutectic solvent driven micellar extractive fermentation of ultrapure fibrin digesting enzyme from *Bacillus subtilis*

**DOI:** 10.1038/s41598-022-04788-w

**Published:** 2022-01-18

**Authors:** Ramya Muniasamy, Bhavani Sowndharya Balamurugan, Devi Rajamahendran, Senthilkumar Rathnasamy

**Affiliations:** Green Separation Engineering Laboratory, School of Chemical and Biotechnology, SASTRA Deemed To Be University, Thanjavur, Tamil Nadu 613401 India

**Keywords:** Analytical biochemistry, Liquid-liquid extraction

## Abstract

Fibrinolytic protease (FLP) is a therapeutic enzyme used in the treatment of thrombolytic diseases. The present study proposed the concept of pH-driven swappable micellar two-phase extraction for the concurrent production and purification of FLP from Bacillus subtilis at cloud point extraction. Extractive fermentation was carried out with a pH swap mechanism and FLP was extracted to the top phase by surfactant deep eutectic solvents (SDES). Shrimp waste was chosen as a sustainable low-cost substrate that yielded a maximum protease of 185 U/mg. Six SDESs were synthesized with nonionic surfactants as hydrogen bond donors and quaternary ammonium salts as hydrogen bond acceptors and their association was confirmed by H^1^ NMR. Thermophysical investigation of the synthetic SDES was accomplished as a function of temperature. Response surface methodology for extractive fermentation was performed with the concentration of SADES (35% w/v), Na_2_SO_4_ (15% w/v) and pH (6.3) as variables and the enzyme activity (248 IU/mg) as a response. Furthermore, purification using gel filtration chromatography was used to quantify the amount of enzyme obtained in the extraction phase (849 IU/ml). After final purification with an anion exchange column, the maximum purity fold (22.32) with enzyme activity (1172 IU/ml) was achieved. The in-vitro fibrinolytic activity has been confirmed using a fibrin plate assay.

## Introduction

Intravascular thrombosis is described as a pathophysiological disorder in which excessive accumulation of blood clots (thrombus) occurs throughout the blood vessels. This chronic condition obstructs blood flow to numerous internal organs and has been reported to be the major cause of cardiovascular abnormalities worldwide^1,2^. Conventionally, recombinant therapeutic enzymes such as streptokinase and urokinase are being used widely for treating these thrombotic abnormalities. Due to their undesirable side effects such as gastrointestinal bleeding and less fibrin specificity^3,4^ intense research has been directed at finding alternative microbial fibrinolytic enzymes with better task specificity. Recent investigations have identified *Bacillus subtilis* as a promising source for the production of specific Fibrinolytic Proteases (FLPs) that are reported to be safer for therapeutic use^5,6^. However, purification of these therapeutically significant enzymes was accomplished with a sequence of downstream operations such as ammonium sulfate precipitation, ultrafiltration, ion exchange chromatography^7^ and affinity chromatography^8^. Although these conventional methods have been adapted on large scale, the addition of subsequent unit operations for enhanced purity makes the process laborious and elevates the cost of the product^9^. Furthermore, the harsh environment involved in some purification steps are reported to have an adverse impact on the native state of the product incurring extensive loss of active enzyme. Therefore, a sustainable and biocompatible purification process that overcomes these limitations is under delve in the modern scientific community.

This delve for alternative purification methods could be readily satisfied by employing extractive fermentation where simultaneous purification of the product from fermentation broth is administered with task specific solvents^10^. Extractive fermentation has been recognized as the most sustainable alternative for enzyme purification in recent decades owing to its advantage of in-situ product recovery^11^. The extraction of products into the solvent-rich phase due to its affinity to the solvent infiltrates the microbial cells and substrates into the raffinate phase making it a superior technique compared to conventional separation methods^12–15^. Earlier investigation reported the usage of neoteric solvents such as ionic liquids and deep eutectics as the most effective phase-forming components in the purification of therapeutic enzymes^16–19^. However, the hydrophilicity of these solvents alleviates the demand for higher concentrations, posing potential challenges in making the process viable on a large scale^20^. These restrictions were mitigated with the use of micellar based extraction systems with selective solvents involving surfactants. Earlier investigation was carried out by Luis et al. for the selective purification of alkaloid^16^ prodigiosin using Triton X 114 resulting in enhanced recovery (81%) of the active enzyme^12^. Effective recycling of primary phase forming components (Triton X 100 and xylitol) post lipase extraction from pumpkin seeds was previously investigated earlier by Amid et al.^21^. Sustainable purification of pectinase^16^ by micellar extraction has been reported. Silvia et al. investigated the extraction of organic and inorganic arsenic using Triton X 100 in combination with ionic choline chloride. The nonionic surfactant Triton X 100 with the ionic cation choline became an attractive phase-forming component in the industry with low cost and commercial availability^22^.

**Figure 1:**
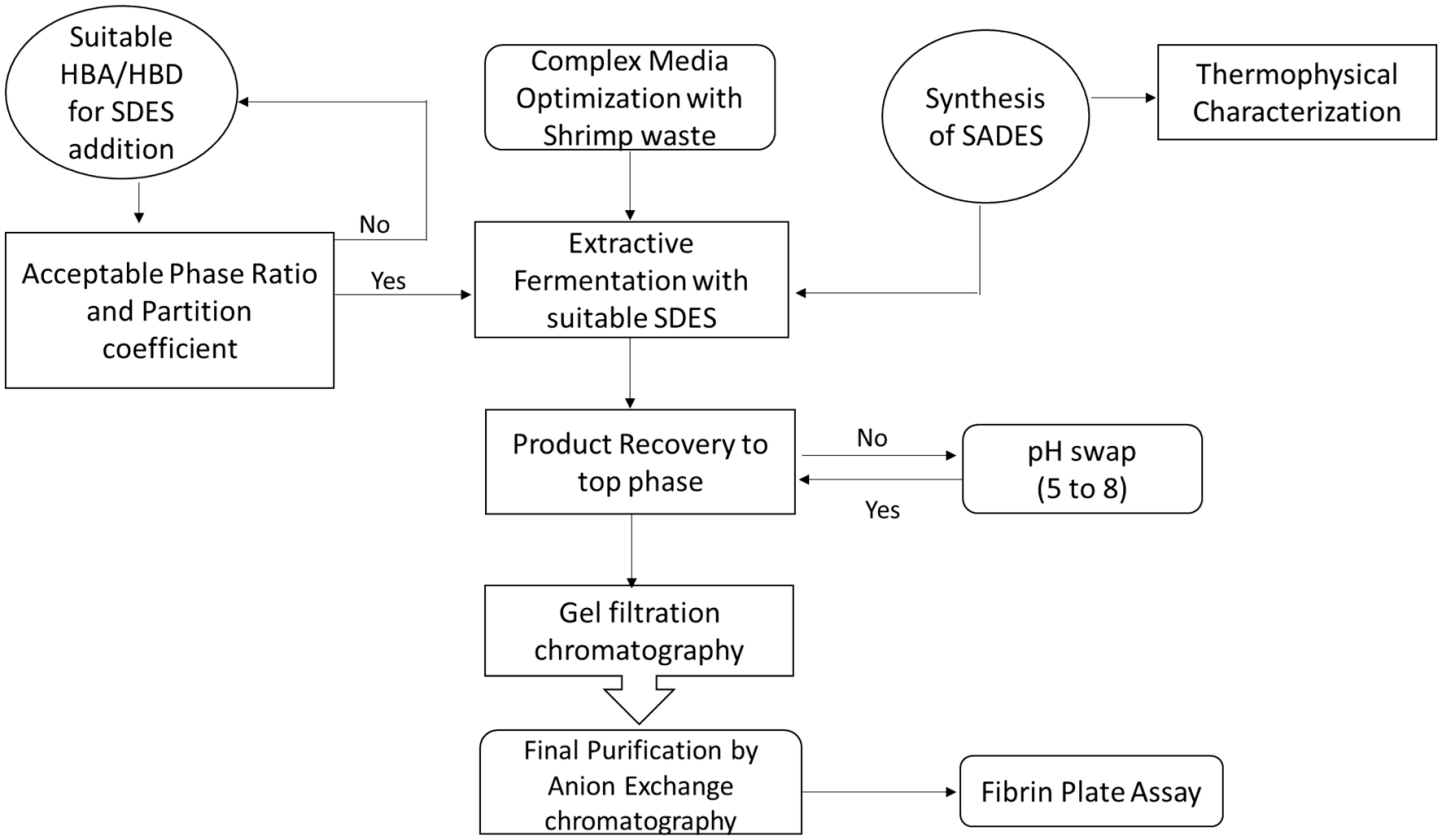
Schematic algorithm of swappable pH extractive fermentation for selective purification of fibrinolytic protease using surfactant based deep eutectic solvents (SDES).

In the current investigation, one-step production and recovery of therapeutic FLP by micellar extractive fermentation was carried out. Six different surfactant deep eutectic solvents (SDESs) were synthesized and their thermophysical parameters were evaluated. These SDESs were then added at various concentrations to the fermentation broth along with various salts to enable aqueous two phase formation. The phase ratio and partition coefficient of individual combinations were determined, and SDES with enhanced enzyme recovery was employed to selectively concentrate fibrinolytic protease on a large scale. Back extraction was accomplished with low concentration of potassium chloride and the enzyme rich fraction was partially purified by size exclusion chromatography. The enzyme fraction from size exclusion chromatography was loaded A fibrin plate assay was used to confirm the fibrinolytic activity of the resulting target enzyme. Figure 1, a schematic representation elucidating the entire work.

## Materials and methods

### Chemical and reagents

Triton X 100 (9002-93-1), choline chloride (67-48-1), tetrabutylammonium bromide (1643-19-2), tetrabutylammonium chloride (1112-67-0), bovine serum albumin (A7030), and Tween 80 (9005-65-6) were purchased from Sigma-Aldrich with 99.9% purity. Peptic digests of animal tissue, yeast extract, Na_2_SO_4_, MgSO_4_, NH_4_Cl, (NH_4_)_2_SO_4_, K_2_HPO_4_, CaCl_2_, Na_2_CO_3_, and Luria Bertani broth media were procured from Himedia, India with < 95% purity (Table 1).

**Table 1:** Various non-ionic surfactants acting as HBD and quaternary ammonium salts acting as HBA involved in formation of six SDES and their corresponding molar ratio for synthesis.

Non-ionic surfactant (HBD)	Cas no	Quaternary ammonium salts (HBA)	Cas no	Supplier	Molar ratio
Triton X 100 (Sigma-Aldrich)	9002-93-1	Choline chloride	67-48-1	Sigma-Aldrich	1:1
		Tetra Butyl Ammonium Bromide	1643-19-2	Sigma-Aldrich	1:1
		Tetrabutylammonium chloride	1112-67-0	Sigma-Aldrich	1:1
Tween 80 (Sigma-Aldrich)	9005-65-6	Choline chloride	67-48-1	Sigma-Aldrich	1:1
		Tetrabutylammonium Bromide	1643-19-2	Sigma-Aldrich	1:1
		Tetrabutylammonium Chloride	1112-67-0	Sigma-Aldrich	1:1

Relative uncertainty for the molar mass of HBA and HBD is u_r_ (X) = 0.2.

### Screening and complex media preparation

*Bacillus subtilis* strain No: 441 was purchased from MTCC, Chandigarh. The purchased strain was plated on skimmed milk agar, and incubated at 37 °C for 24 h. The colony with maximum proteolytic activity (forming a clear zone in skimmed milk agar) was subcultured on skim milk agar to enhance protease activity^23^. The pure colony thus isolated was used to inoculate the seed culture in nutrient broth (5.0 g/L), yeast extract (1.5 g/L), and sodium chloride (5.0 g/L)) and incubated overnight^24^.

Various complex sources such as groundnut cake, cottonseed cake, and shrimp wastes were purchased from the local market, sterilized with 0.1% sodium hypochlorite solution and dried in a hot air oven. These dry complex sources were then macerated with 20 mM Tris–HCl buffer and centrifuged at 10,000 rpm. The supernatant thus obtained was considered as a complex substrate (for enzyme production) and was stored at—4 °C for further use. The production media was prepared by mixing the complex substrates (1% v/v) with glucose (10 g/L), NH_4_Cl (3 g/L), MgSO_4_ (2 g/L), Na_2_CO_3_ (0.6 g/L), CaCl_2_.2H_2_O (5 g/L) at pH 7.2 and sterilized at 121^0^ C for 15 min as described by Cho et al.^25^. The sterile medium was then inoculated with 1% overnight grown seed inoculum and incubated for 21 h at 120 rpm.

### Synthesis and thermophysical characterization of surfactant based deep eutectic solvents (SDESs)

**Figure 2:**
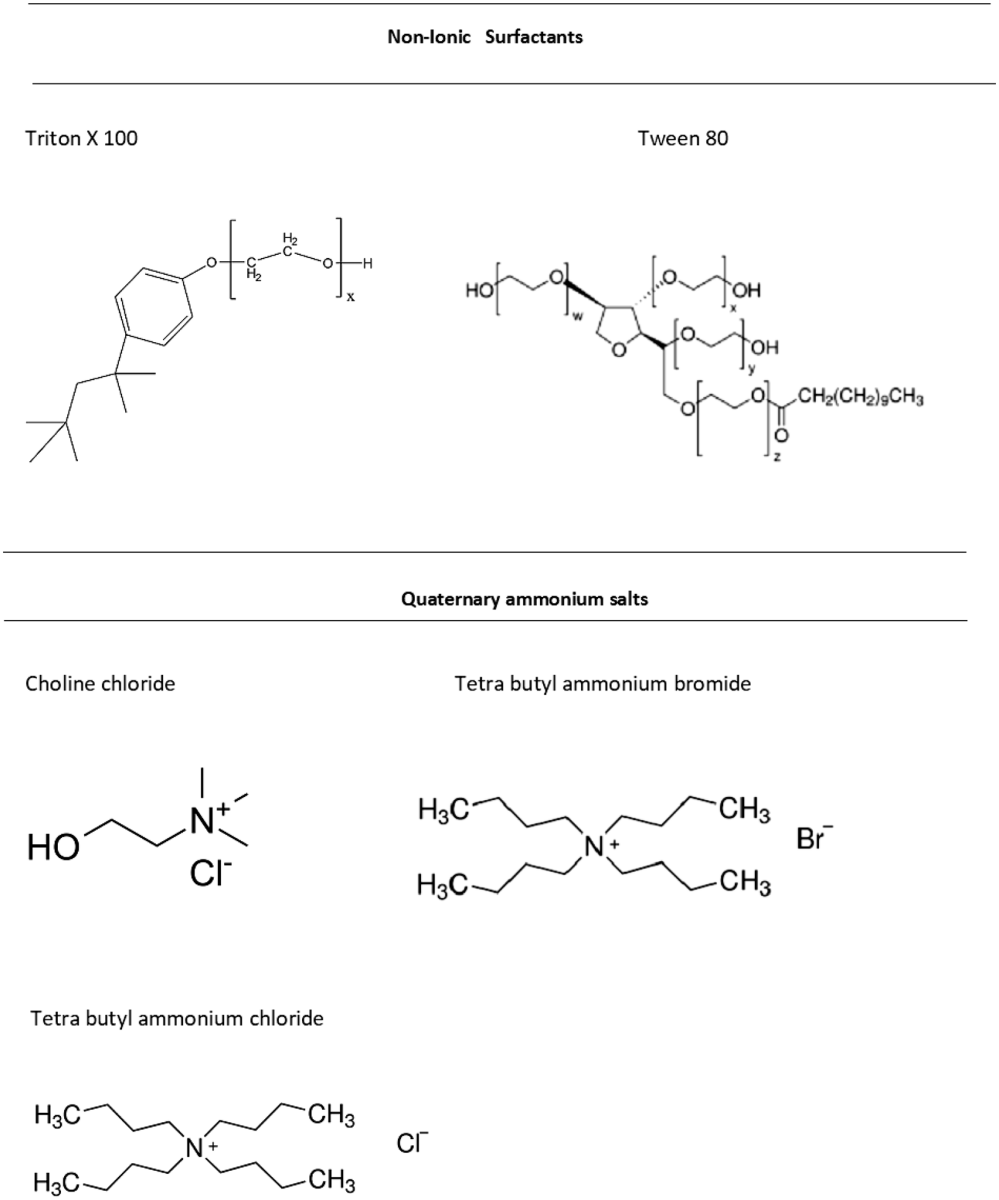
Structures of non-ionic surfactants acting as HBD and quaternary ammonium salts acting as HBA in formation surfactant based deep eutectic solvents (SDES).

The present study involved the synthesis of six surfactant-based DES (SDESs) with Triton X 100 and Tween 80 as hydrogen bond donors (HBDs) and ammonium salts such as choline chloride, tetrabutylammonium chloride, and tetrabutylammoniumbromide as hydrogen bond acceptors (HBAs) (Fig. 2). Individual SDES were synthesized by mixing both HBA and HBD in a defined molar ratio (1:1 and heating the mixture at 80 ^0^C with continuous stirring. The molar ratio of the HBD: HBA depends on the density of the compound. So when 1:1 molar ratio was used the density was moderate which lead to the formation of two phases. The ratio also depends on the exact balance between the cation and anion as reported by Renita et al.^26^. The resulting dense and transparent liquid is described as a eutectic mixture. The stability of the SDES solution thus formed was observed for 45 days at room temperature in a vacuum desiccator to avoid moisture entrapment. The molar composition of all six SDES along with their abbreviations is depicted in Table 3.

The density, viscosity, and refractive index of all SDESs formed were measured in the temperature interval of 273–323 K (Table 4a, b, c). A Rudolf digital density meter (DDM 2910) with an in-built thermocouple for temperature control was used to measure the density of all eutectic mixtures. The viscosity of all SDESs was quantified using Brookfield DV II + Digital Viscometer. The thermal jacket attached to the viscometer was regulated by an external oil bath. The refractive index of all eutectic mixtures was calculated by Atago (MASTER-PM) hand-held refractometer along with the sodium D1 line. All instruments used were calibrated with deionized water before measurement.

The H^1^ NMR of all SDES was determined with a 300 MHz BRUKER AVANCE II Spectrophotometer equipped with a 5 mm BBO probe. ~ 8 mg of SDES was dissolved in 500 µl of D_2_O in the quartz NMR tubes and ultrasonicated for 5 min. Resonance at 298.18 K was observed and progress was recorded with the sequence library Topspin 3.2. (Bruker biospin, Switzerland).

### Binodal curve determination of SDES

10 ml of aqueous SDES (80% (v/v)) was added to a fresh separation funnel and aqueous sodium sulfate (20% (v/v)) was added dropwise until a cloudy appearance in the transparent solution. The milky solution was left undisturbed to enable the formation of two distinct phases. The phases were separately collected and the mass of individual phases was noted by a Shimadzu BL-220H weighing balance. The procedure was repeated after diluting the earlier cloud point into the homogenous monophasic solution by dropwise addition of water until the extract volume gets minimal. The mass fractions of SDES and salt observed in each cloud point were plotted individually on the X and Y axes correspondingly to obtain the binodal curve of SDES as described by Capela et al.^27^.

### Micellar based extractive fermentation (MEF) of fibrinolytic protease

Extractive fermentation was carried out on a 14 h batch culture (culture in log phase of production) to obtain an effective yield of fibrinolytic protease. In a batch experiment, sterile SDES (80% w/v) and salt (20% w/v) were weighed and added to the broth and vigorously agitated until the formation of the cloud point. The pH of the milky heterogeneous mixture was altered from 7.2 to 6 by adding 0.1 M HCl aseptically and the content was left undisturbed until the formation of two distinct phases. Once separation was achieved with a well-defined boundary, individual phase components were aspirated carefully without disturbing the intermediate layer. The total volume of the system and the corresponding individual volume of the two phases were noted. The phase ratio of individual SDES and salt combinations were evaluated with the following equation:

1 $$P_{r} = \frac{{V_{top} }}{{V_{bottom} }}$$

where P_r_ denotes the phase ratio, V_top_ denotes the volume of the top phase and V_bottom_ denotes the volume of the bottom phase respectively.

The enzyme recovered in individual phases was determined by its corresponding fibrinolytic activity as described by da silva et al.^28^. The partition coefficient (K) of the system was determined as the ratio between the corresponding concentrations of fibrinolytic protease in the top and bottom phases. The selectivity of individual SDESs toward efficient recovery of the enzyme could be determined with the partition coefficient values. The partition coefficient K was determined by

2 $$K = \frac{{C_{top} }}{{C_{bottom} }}$$

where K = partition coefficient, C_top_ = concentration of protease in the top phase (mg/ml), and C_bottom_ = concentration of protease in the bottom phase (mg/ml).The purity factor (P) of the enzyme recovered in the top phase was interpreted as the ratio of FLP activity in the top phase to the corresponding activity in the crude phase

3 $$P = \frac{{\frac{{C_{top} }}{{T_{top} }}}}{{\frac{{C_{crude} }}{{T_{crude} }}}}$$

where P = purity factor, T_top_ = total protein concentration in the top phase (mg/ml), C_crude_ = fibrinolytic protease concentration of the crude extract (mg/ml), T_crude_ = total protein concentration of the crude extract (mg/ml). The yield (%) of fibrinolytic protease (Y_flp_) was calculated as the proportion of fibrinolytic activity in the top phase to the fibrinolytic activity in the initial crude in percentage.

4 $$Y_{flp} = \left( {\frac{{C_{top} }}{{C_{crude} }}} \right)*100$$

The specific activity (S) of the enzyme was calculated by the ratio of protease activity in the top phase to the total protein in the crude.

5 $$S = \frac{{C_{top} }}{Tcrude} .$$

### Optimization of micellar extractive fermentation by response surface methodology

The influence of essential components on the effective recovery of fibrinolytic protease by micellar extractive fermentation was determined by response surface methodology. The statistical significance of individual variables on the recovery of protease was determined using a central composite design (CCD) with the help of Design expert (v10.1 Stat-ease, Minneapolis USA). The SDES concentration (25–45% v/v), K_2_HPO_4_ concentration (5–15% w/v), and pH of the system (4.5–7.5) were the most influential factors chosen for optimization. The fibrinolytic activity in the SDES-rich top phase was selected as the response factor. A set of 32 experiments with 6 center points, 2 axial and a radial point were formulated. Each trial was conducted in a 250 ml flask filled with100 ml of fresh culture media. A 1% v/v seed culture was inoculated, incubated at 40 °C and 150 rpm, and subjected to batch fermentation for 14 h. Following incubation, aqueous solutions of SDES and K_2_HPO_4_ were added at concentrations corresponding to the respective trials and left undisturbed until the formation of two distinct phases. The amount of enzyme recovered from both phases was determined by fibrinolytic activity determination.

### Back extraction of SDES

The fibrinolytic protease-rich SDES phase was back-extracted to recover the enzyme in its native form. The SDES rich phase recovered from the previous step was transferred to a sterile flask and an equal volume of fresh aqueous potassium chloride solution (15% W/V) was added and incubated with reduced agitation^29^. The vander wall’s force between the SDES and enzyme became destabilized leading to the infiltration of active enzymes to the salt rich phase. The excess water in the SDES recovered was removed and the solvent was reused for a fresh batch of extraction cycles. The amount of SDES recovered (R_SDES_) was determined as detailed by Mehrrnoush et al. using the following Eq. (6).

6 $$R_{SDES} = \frac{{A_{final} }}{{A_{initial} }} *100$$

where A_final mass_ indicates the mass of SDES recovered and A_initial mass_ is the total mass of SDES introduced into the system.

### Biodegradability of SDESs

Individual SDES was prepared as a solution of 10 mg/l with mineral media incubated with water at 1.0 ml concentration. The control was only water sample without any SDES. The reference was sodium benzoate. Incubation as kept at 298 K in dark atmosphere for 30 days. The biological oxygen demand was measured every 10 days as reported by Zhao et al.^30^.

### Preparative purification of fibrinolytic protease by size exclusion chromatography

A 5 ml Sephadex G-15, Size Exclusion chromatography column (Akta Prime plus) was used for the preparative purification of the recovered enzyme. Prior sample injection, 20 mM phosphate buffer at pH 7) with a flow rate of 2 ml/min (flow rate maintained constant during the process) was used for column equilibration. The crude extract obtained from the back extraction step was loaded and elution of the protein molecules was achieved by passing the same equilibration buffer^31^. Individual fractions showing the peak observed in the chromatogram were collected and eluent showing maximum fibrinolytic activity were designated as the fraction of interest. The efficiency of the column toward separation of fibrinolytic protease from other proteins was determined using the number of theoretical plates per meter (NTP).

7 $$NTP = 16\left( {\frac{{t_{r} }}{{W_{b} }}} \right)^{2}$$

where NTP = Number of theoretical plates, t_R_ = retention time; W_b_ = peak width. Similarly, the binding efficiency was determined by calculating the retention volume with the following equation.

8 $$R_{v} = R_{t} *W_{b}$$

where R_V_ = retention volume, R_t_ = retention time, W_b_ = base width of the peak.

### Ultra purification by DEAE-anion exchange chromatography

The eluent fraction obtained through preparative purification was ultrapurified with a 5 ml DEAE Sepharose anion exchange chromatography column (GE, AKTA prime plus).The column was equilibrated with 20 mM Tris–HCL, pH 8.2 buffer until zero baseline was achieved. Then the sample was loaded through the sample port which entered the column along with the equilibration buffer^32^. All undesirable molecules did not bind to the column were removed with an equilibration buffer. Elution was accomplished by passing the elution buffer 50 mM Tris–HCL, 1 M NaCl, pH 8.5 as a gradient along with equilibration buffer. Due to a change in the buffer ionic strength, the target enzymes bound to the matrix were eluted serially depending on their binding strength to the column matrix. The fraction exhibiting the highest enzyme activity was regarded as the ultrapure enzyme fraction. The yield of the ultrapure enzyme fraction was calculated with the following equation.

### Fibrin plate assay

The fibrinolytic activity of the ultrapure fraction of enzyme obtained from anion exchange chromatography was measured using the fibrin plate degradation method as mentioned by cruz et al.^13^. The fibrin gel was prepared by adding 10 ml of fibrinogen (0.5% w/v) along with 0.1 ml of thrombin (50 NIH U/ml) into a 15 ml agarose gel (agarose 1% w/v) and allowed to solidify in a petri plate. A hole of 3 mm diameter was punctured on the gel and loaded with 10 µl of crude and ultrapure fractions of enzymes. The plate was incubated for 12 h at 37 °C and the zone of clearance with fibrin degradation was measured carefully.

## Results and discussion

### Effect of complex media on FLP production

Substrate utilization is one of the foremost cost-defining factors in the production of therapeutic enzymes. Some of the underutilized residuals were reported to be used as potential substrates for the large scale production of commercial and therapeutic enzymes. *B. subtilis* is the most promising bacteria for the production of fibrinolytic protease with the optimal yield varying with the type of complex substrate supplied^33^. Among the various nitrogen sources, the maximum yield of protease (185 U/mg) was observed to occur with shrimp waste as the primary nitrogen source compared to groundnut cake and cottonseed cake with moderate enzyme production of 100 U/mg and 110 U/mg respectively (Table 2). This might because shrimp waste is rich in essential minerals such as sodium, potassium, manganese and iron along with a vast reserve of proteins and fats that aids in extensive secondary metabolite production^34^. It is also capable of metabolizing its nitrogen content slowly into the media which favors the maximum production of protease enzymes^35^. On the other hand, groundnut cake and cottonseed cake could not sustain the release of nitrogen and were proven incapable of acting as potential nitrogen sources for effective enzyme yields. An identical investigation carried out by Lang et al. concluded that shrimp shell powder acts as a potential inducer of alkaline protease production from *Bacillus subtilis*^36^. Similar investigations on the production of fibrinolytic protease with various complex substrates such as soybean filtrate^37^, fodder yeast^5^ and Cassava starch^38^ have comparatively yielded lower amounts of enzyme than shrimp waste.

**Table 2:** Various nitrogen sources as substrate used as a complex media and it enzyme activity.

S. nos.	Nitrogen sources	Enzyme activity (EA) (U/ml)
1	Groundnut cake	100
2	Shrimp waste	185
3	Cotton seed cake	110

Relative uncertainty for the enzyme activity is u_r_ (EA) = 0.02 U/ml.

### Thermophysical characterization of SDES

Surfactant-DES synthesized by combining Triton X 100, Tween 80 (Nonionic surfactants) with quaternary ammonium salts choline chloride, tetrabutylammonium bromide, and tetrabutylammonium chloride (DES 1—TX: CCL, DES 2—TX: TBAB, DES 3—TX: TBAC, DES 4—TW: CCL, DES 5—TW: TBAB, DES 6—TW: TBAC) was heated in the appropriate molar ratio and remained homogenous at room temperature. This exposed that the SDES was capable of effective extraction of therapeutic enzymes and was well established as a micellar-based aqueous two-phase system.

#### Density

**Figure 3:**
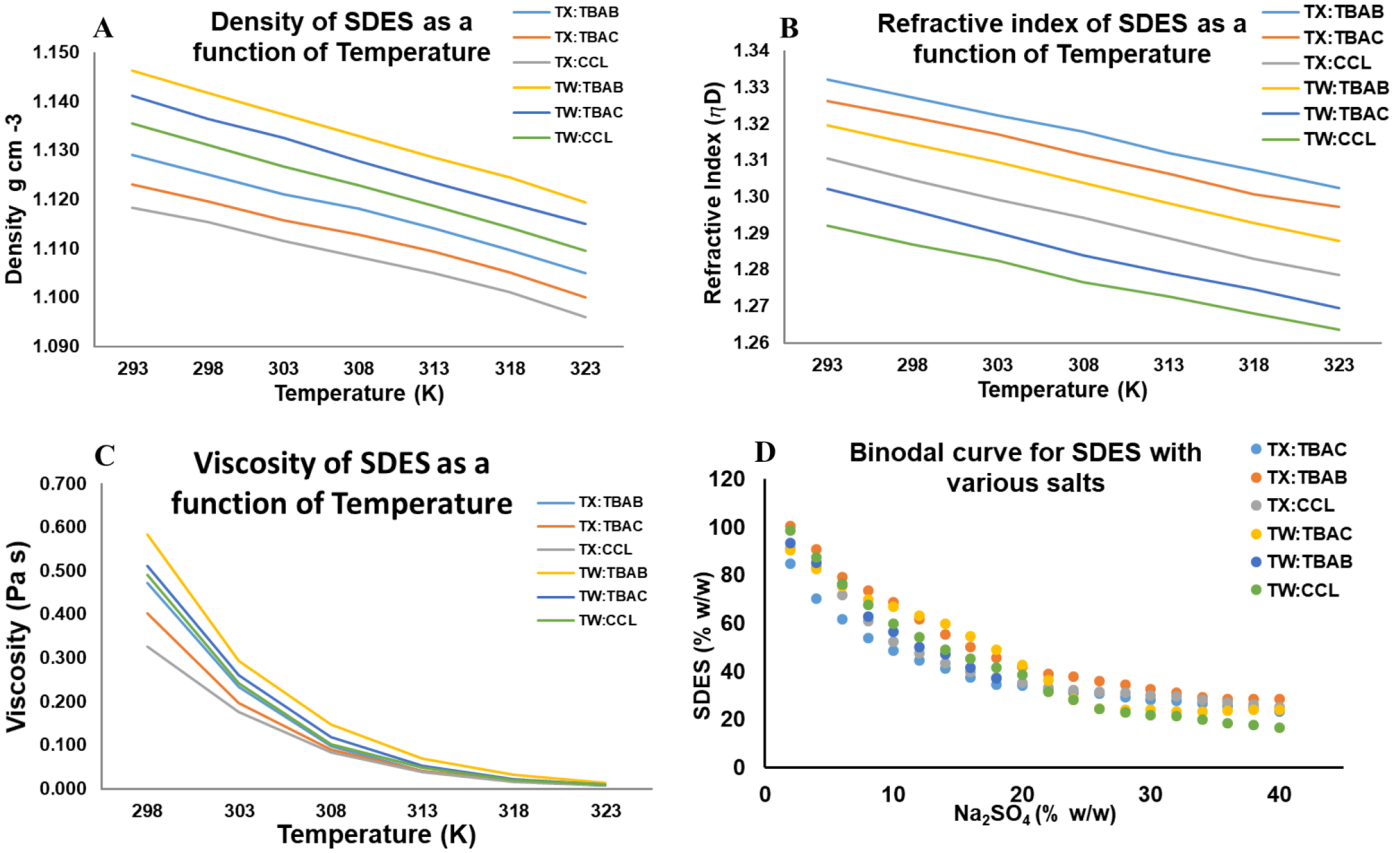
Density (**A**), Refractive index (**B**) and Viscosity (**C**) of different SADES subjected to temperature in the range of 293–323 K and (**D**) Binodal curve determination of various SADES with sodium sulphate.

The density of the synthesized SDES was measured as a function of temperature in the range of (293–323) K. From Fig. 3A, it was evident that a linear increase in density was observed with a proportional decrease in temperature and increase in the molar ratio of DES. As the temperature increases, the intermolecular interaction between the corresponding HBA and HBD becomes less dense due to thermal expansion^39^. The highest density was recorded for DES 5 (1.146 g cm^−3^) and the lowest was recorded for DES 1 (1.024 g cm^−3^) at 323 K. The order of density at both extremities of temperature was found to be identical as follows DES 1 < DES 3 < DES 2 < DES 4 < DES 6 < DES 5. The extraction of the fibrinolytic protease was observed to be critically influenced by the density of the corresponding SDES under investigation. The lowest molecular weight of ionic choline resulted in a lower density SDES with both Triton and Tween. Tween interaction were found to be more effective with quaternary ammonium salts, resulting in a higher density compared to Triton^40^. Denser eutectic mixtures tend to accumulate in the top phase, virtually leaving limited space for the enzyme, resulting in a lower yield of product. In contrast, SDES with low density consumes a large amounts of salt, forming a biphasic system with a low phase ratio and leading to infiltration of a large amounts of enzyme to a salt rich phase. Therefore, it could be conclusive from the above postulations that SDES with moderate density (DES 1) acts selectively towards effective extraction of fibrinolytic protease. A similar investigation using a hydro low transition-temperature mixture for the biomass pretreatment of lignin concluded effective product recovery using mixtures with moderate density^41^.

#### Refractive index

The refractive index of the synthesized SDES was observed to decrease linearly with increasing temperature. The extraction of the DES was improved with a lower refractive index. The refractive index value of DES 6 was found to be the highest (1.34) while the DES 1 mixture had the lowest value (1.292). The intermolecular cohesive energy between HBA and HBD tends to be higher for larger molecular weight surfactants resulting in SDES with a high refractive index. On the other hand, lower molecular weight surfactant counterparts form SDES with a low refractive index due to the scarcity of hydrogen bonding between the corresponding HBA^42^. SDES with a higher refractive index is observed to have an elevated molar volume and thus acts inert while salting out with minimal extraction efficiency. In contrast, SDES with a low refractive index and fewer intermolecular interaction has a low salting out volume, providing a minimum partitioning effect. Therefore, SDES with a moderate refractive index is preferable because it provides an improved free volume for the target molecule, enhancing the extraction with a better salting out coefficient. The decreasing order of refractive index was as follows: DES 5 > DES 6 > DES 1 > DES 2 > DES 3 > DES 1 (Fig. 3B).

#### Viscosity

The viscosity of all SDESs was measured in relation to temperature in the range of 293–323 K. It could be observed that the viscosity of all the mixtures decreased in a non-linear fashion as the temperature increased. This is because of the reduction in noncovalent interactions between HBA and HBD in the SDES at higher temperature decreasing the surface tension of the mixture^43^. Similar to investigation performed by Gajardo et al., the molecular weight of the corresponding HBA and HBD was observed to have a substantial influence even on the viscosity of the mixture^44^. Surfactants (Tween 80) with high molecular weights associate more strongly with the quaternary ammonium salts (0.583 Pa.s), making the resultant SDES more viscous than their low molecular weight counterparts (Triton X 100) (0.327 Pa.s). Additionally, viscous DESs have low product retention capacity due to their inability to generate void space for target molecule accommodation. DESs with low viscosity exhibit poor salting out capability resulting in poor phase formation^45^. Therefore, SDES with moderate viscosity is assumed to act selectively in concentrating fibrinolytic protease to the top phase. The viscosity of the SDES under investigation follows the order of DES 1 < DES 3 < DES 2 < DES 1 < DES 6 < DES 5 (Fig. 3C). An identical investigation carried out by Wang et al. for choline chloride-based DESs concluded that the addition of glycerol as an HBD results in higher viscosity than the ethylene glycol counterparts. This is due to the additional hydroxyl group in glycerol enabling a stronger binding strength between HBA and HBD, thus resulting in a deviation in its physical properties^36^.

#### Binodal curve determination of SDES

The critical concentrations of both SDES and salt were observed to have greater significance in two phase formation and product retention. This effect could be elucidated with binodal curve determination by the cloud point titration method^38^. The binodal curve between the individual SDES under investigation and its corresponding salt is shown in Fig. 3D. The extended curve obtained for high-density SDES signifies its excess salting out capability making them suitable for liquid–liquid extraction applications. In contrast, low-density SDES requires less salt to achieve consecutive cloud points resulting in a narrow biphasic region. The SDES with reasonable density is observed to have sufficient salting-out volume, resulting in a large two-phase region. The stability of the biphasic system was influenced by the entropy change of SDES and salt combination. The phase formation for the SDES and salt occurred in the following order DES 3 > DES 4 > DES 3 > DES 1 > DES 6 > DES 5.

#### H^1^ NMR analysis of synthesized SDES

Analysis of H^1^ NMR analysis of the synthesized SDES provides the details of the interaction between both HBA and HBD that results in the formation of the corresponding eutectic mixture. The distributed occurrence of peaks in the graphs (Supplementary file: Figs. S1–S6) signifies the native state of HBA and HBD with certain indefinite peaks denoting the noncovalent association between them. In addition, the indeterminate sharp peaks distributed throughout signify that the noncovalent associations that occurred at the eutectic temperature are strong making the mixture stable at room temperature.

### Effect of SDES on fibrinolytic protease extraction

**Figure 4:**
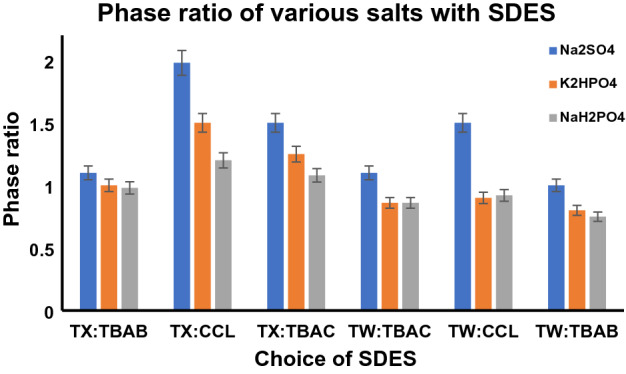
The partition coefficient values of various salts in combination with all six SDES under investigation.

Each phase composition was determined by titrating all SDES and salt concentrations (Table 2). The nonionic surfactant Triton X 100 with choline chloride (DES 3—TX: TBAC) (1:1)) as HBD and HBA, respectively was proven to be effective in the extraction of therapeutic fibrinolytic protease into the SDES-rich top phase compared with other counterparts. The specificity of SDES in isolating the enzyme was greatly influenced by the amount of protease present in broth. Denser SDES exhibits a lower partition coefficient, as the void space for accommodation of the protease in the top phase is limited, enabling its infiltration into the bottom phase. A lower density of SDES requires more salt to enable two phase formation as a result of the low salting out ability leading to low volume and partition coefficient. SDES with moderate density improved the extraction of FLP into the micellar rich phase with better partition coefficient of 2.17 (Table 3). The nonionic surfactants Tween 80 and Triton X 100 enhance the enzyme activity at higher concentrations compared to ionic surfactants^46^. The partition coefficient follows the descending order DES 1 < DES 3 < DES 2 < DES 4 < DES 6 < DES 5 (Fig. 4). A similar investigation by Silva et al. Triton X 100 with choline chloride as the phase forming component required 19 times less ammonium pyrimidine dithiocarbonate as the primary extracting agent for concentrating arsenic than conventional polymer-based ATPSs^22^.

**Table 3:** Selectivity of SDES determined with their corresponding partition coefficient and enzyme activity.

Choice of SDES (70% w/w)	Enzyme activity (EA) U/ml	Partition coefficient (K)
DES 1	12,855	1.56
DES 2	13,958	1.83
DES 3	15,249	2.17
DES 4	13,684	1.34
DES 5	10,658	0.78
DES 6	11,005	0.96

DES 1—TX: CCL, DES 2—TX: TBAB, DES 3—TX: TBAC, DES 4—TW: CCL, DES 5—TW: TBAB, DES 6—TW: TBAC.

Relative uncertainty for the enzyme activity and partition coefficient are u_r_ (EA) = 0.02 U/ml and u_r_ (K) = 0.2.

Salt selection in the cloud point extraction for micellar ATPSs is essential, as it promotes phase separation. The volume ratio of the micellar and aqueous phases is greatly influenced by the salting-out effect^47^. Sodium sulfate, potassium dihydrogen phosphate, and sodium dihydrogen phosphate were investigated as potential chaotropic agents for micellar ATPS formation. Sodium sulfate with moderate ionic strength (salting out) was observed to enhance the two-phase system with a better phase volume and cloud point. The higher ionic strength of inorganic salt forms the phase immediately displays a wider biphase region and does not denature protease. Due to the ionic strength, phase separation has been observed to occur in the order of NaH_2_PO_4_ > Na_2_SO_4_ > KH_2_PO_4_ > which closely follows the Hofmeister series of cations and anions and encourages a salting-out nature.

### Effect of swappable pH at cloud point extraction

Micellar-based ATPSs are formed when a cloudy appearance takes place and the homogeneity of the mixture is disrupted at the critical micellar concentration of salt and SDES^48^. In the current investigation, a swappable pH-assisted micellar aqueous two-phase system was accomplished with TX: TBAC with 80% extraction efficiency. Triton X 100 does not easily ionize in an aqueous solution and with a cloud temperature of 65 °C, functions as a better phase forming alternative at room temperature^49^. The pH of the system affects the partitioning of biomolecules by altering the charge and surface properties of surfactants. When the phase forming components were added to the broth, the protein competed for each other to move into the micellar rich phase based on its hydrophobicity. The greater the hydrophobicity of the protein the greater the affinity toward the micellar-rich DES phase. The swap in pH to 6.3 by addition of 0.1 M HCl, altered the protein charge so that the positive and open structure of the protein became enclosed in the SDES-rich phase, as Yu et al. reported a switchable pH of the aqueous phase to form biphasic from the monophasic region^50^. The recovery of fibrinolytic protease was maximum at optimum pH 6.3 with partition coefficient of 2.17 because the enzyme remained active and stable at acidic pH. The cloud point and micelle size were not affected by pH in the range of 2–10^51^. The decrease in pH with the increase in the temperature until 50 °C favored extraction into the SDES-rich top phase. The extraction at pH 5 made the broth more acidic, and the enzyme lost its stability. At pH 7, an interface layer precipitating the product occurred. At pH 8, the protein was almost equal to its pI which makes the protein a net charge of zero^52^. SDES was considered neutral and the change in pH was not affected as the zwitterion detergent blended with ionic salt formed ambivalent surfactant micelles ^49^. The pretreatment of biomass at less than pH 7 solubilized the hemicellulose fractions by adding HCl or H_2_SO_4_^53^. Switchable hydrophilicity was reported for the extraction of metal ions and organic compounds by bubbling of CO_2_^54^. An identical study was investigated using switchable solvent for the extraction of copper in food, water and hair samples with environmental applications^55^.

### Optimization of micellar extractive fermentation using SDES

**Figure 5:**
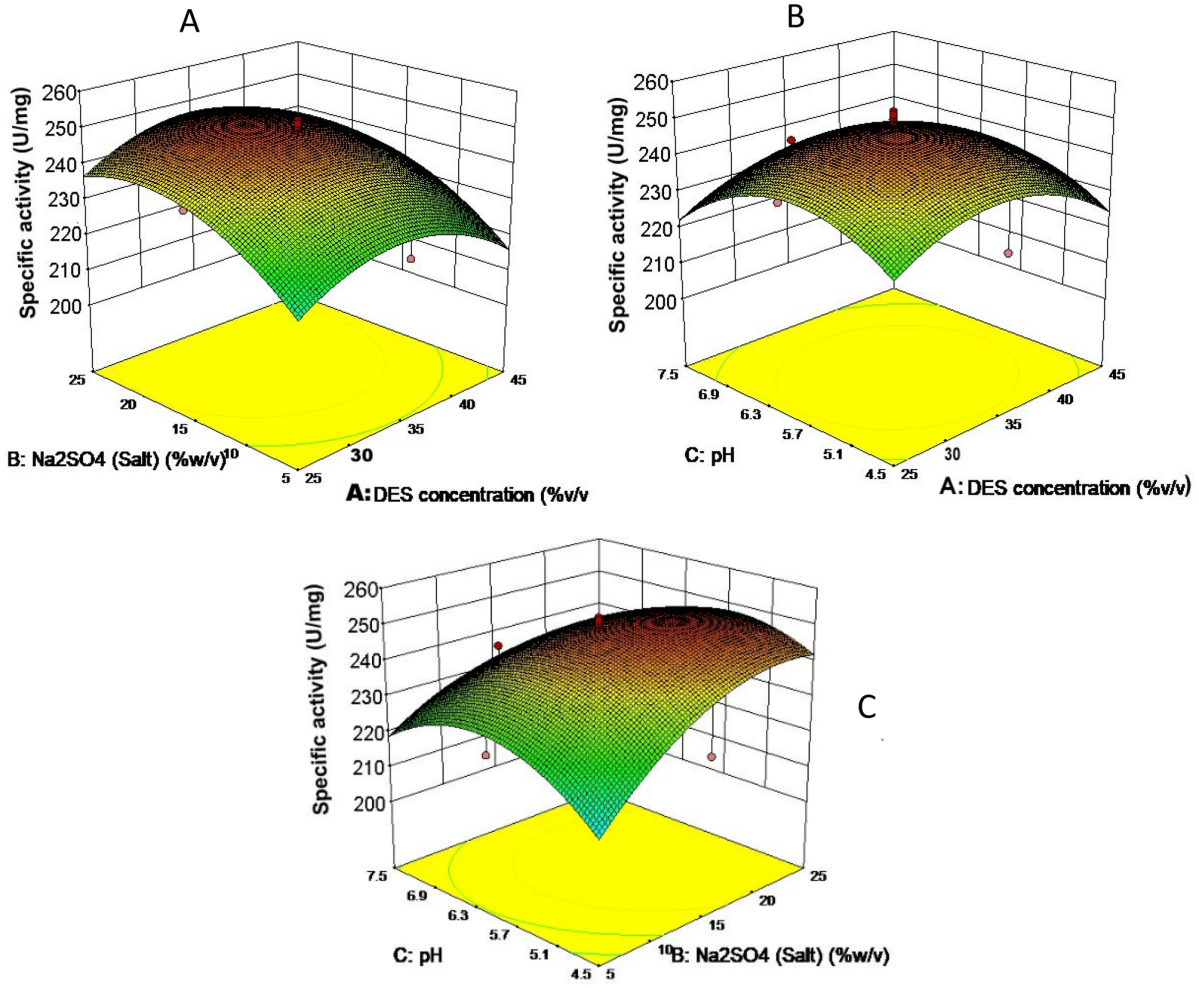
Response surface methodology based optimization of SDES concentration, Salt concentration and pH of medium for effective recovery of fibrinolytic protease. (**A**) Denotes the cumulative effect of DES and salt concentration, (**B**) denotes the cumulative effect of pH and SDES concentration and (**C**) denotes the effect contributed by pH in combination with salt concentration for selective recovery of the enzyme.

All the independent factors that has significant influence on the selective recovery of fibrinolytic protease was chosen for optimization. The specific activity of the resulting enzyme fraction remains distributed between 208 and 245 IU/mg. Notable is the substantial effect of both pH and SDES concentration on the recovery of active enzymes. Furthermore, it could clearly be observed that the reduced concentration of salt has a significant effect on the recovery of enzymes, thus denoting its unsubstituted role in the selective partitioning of fibrinolytic protease. An optimal enzyme activity of 248 IU/mg with purity fold of 22.32 was achieved with Na_2_SO_4_ (15% w/w) and SDES (35% w/w) along with an optimal pH of 6.3 (Fig. 5). The purity fold obtained in this report was greater than the purity fold of pectinase (15.2) investigated by Amid et al. using Triton X and sorbitol for phase separation^21^. A similar study on menthol based natural deep eutectic solvents (NADES) reported purity fold 21.2 for the recovery of fibrinolytic protease^2^.

### Back extraction and recovery of DES

This novel micellar driven ATPS has advantage of recycling of phase forming components. The recovery percentage achieved here is 95% with 15% (W/V) KCL. The salt added to the SDESs rich top phase weakens the interaction between SDESs and target protein leaded the enzymes moved towards the salt rich phase^56^.This recovery percentage indicates that this system may be economical and feasible for industrial applications.

### Biodegradability test

The biodegradability of SDES was observed to be > 80% at the end of 30 days. It was evaluated in comparison with sodium benzoate, detected as 72%^57^. The highest biodegradability was recorded as 92% for DES 3. For other SDESs it was recorded as 89 for DES 1.85% for DES 2.81% for DES 4, 80% for DES 5 and 83.5% for DES 6.

### Preparative purification of FLP by size exclusion chromatography

**Figure 6:**
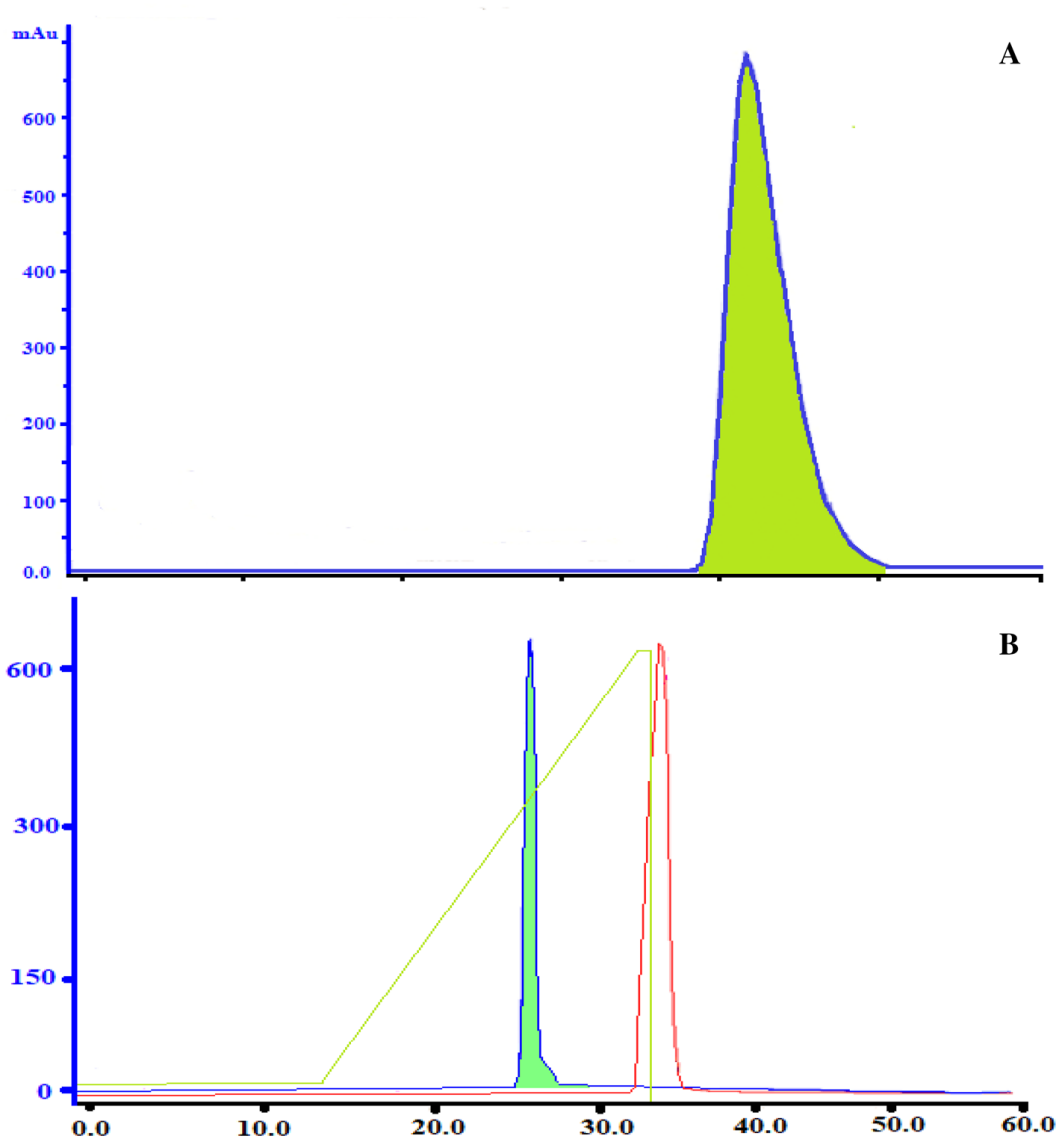
(**A**) Gel filtration chromatogram and (**B**) anion exchange chromatogram obtained through purification of fibrinolytic protease obtained from extractive fermentation using surfactant based DES.

The extractive fermentation sample was loaded on sephadex G-15 for quantification. The separation was based on the molecular size of the molecules. The run was optimized with a 1.0 ml/min sample flow rate, which gave the sample adequate time to interact with the stationary phase. The resolution of the column was increased when the flow rate was reduced and reflected on the plate numbers. The peak eluted fraction retention time was 10 min, with a maximum activity of 849 IU/ml (Table 4). The retention volume and number of theoretical plates were estimated to be 24 ml and 3.2 NTU. The eluted fraction with maximum activity (Fig. 6A) was collected and further purified using a DEAE anion exchange column.

**Table 4:** Measurement of density, viscosity and refractive index at various temperature.

Temp (K)	DES 1	DES 2	DES 3	DES 4	DES 5	DES 6
**(a) Density (ρ) at different temperature**						
293	1.1183	1.1294	1.1231	1.135	1.1463	1.141
298	1.1153	1.1251	1.1196	1.131	1.1417	1.136
303	1.1115	1.1211	1.1158	1.127	1.1373	1.133
308	1.1083	1.1181	1.1129	1.123	1.1329	1.128
313	1.1049	1.1141	1.1093	1.119	1.1286	1.123
318	1.1011	1.1097	1.1051	1.114	1.1246	1.119
323	1.0961	1.1049	1.1001	1.11	1.1194	1.115
**(b) Viscosity (µ) at different temperature**						
298	0.327	0.473	0.403	0.491	0.583	0.512
303	0.176	0.233	0.197	0.242	0.294	0.261
308	0.083	0.098	0.089	0.103	0.147	0.118
313	0.038	0.041	0.04	0.049	0.07	0.052
318	0.016	0.019	0.018	0.02	0.032	0.021
323	0.007	0.008	0.009	0.0093	0.013	0.009
**(c) Refractive index (RI) at different temperature**						
293	1.311	1.332	1.326	1.292	1.32	1.3021
298	1.305	1.327	1.322	1.2869	1.314	1.2963
303	1.299	1.322	1.317	1.2824	1.31	1.2901
308	1.294	1.318	1.312	1.2765	1.304	1.2841
313	1.289	1.312	1.306	1.2727	1.298	1.2791
318	1.283	1.307	1.301	1.2681	1.293	1.2746
323	1.279	1.302	1.297	1.2635	1.288	1.2695

*(a) Standard uncertainty is u (T) = 0.1 K (0.63 confidence level) and expanded uncertainty of density is U (ρ) = 0.02 gcm^−3^.

*(b) Standard uncertainty is u (T) = 0.1 K (0.63 confidence level) and expanded uncertainty of density is U (µ) = 0.2 kgm^−1^ s^−1^.

*(c) Standard uncertainty is u (T) = 0.1 K (0.63 confidence level) and expanded uncertainty of density is U (RI) = 0.2.

### Ultra purification of fibrinolytic protease with anion exchange chromatography

The DEAE- Sepharose anion exchange column was equilibrated with the desired pH (8.2) and ionic strength (equilibration buffer) so that the column was prepared for binding or adsorption of the target molecules. The sample was injected at a flow rate of 1 ml/min dissolved into the column, where the molecules with suitable charge displaced the counter ions in the column and bound to it. Then, a washing step was performed with equilibration buffer to remove the unbound molecules. The next step was elution accomplished by an elution buffer by increasing the ionic strength (1 M NaCl) and altering the pH (8.5). Desorption of target molecules was achieved by altering the pH with increasing salt concentration, and the molecules eluted out based on the binding strength. The eluted fraction with a retention time of 10 min was collected and the fibrinolytic protease activity was found to be 1172 IU/ml (Table 5). The recovery of fractions was found to be 90% (Fig. 6B).

**Table 5:** Increase of purity fold corresponding to various affinity separation operations carried out and their effect on the yield and specific enzyme activity of fibrinolytic protease.

Purification steps	Total protein (TP) mg/ml	Enzyme activity (EA) U/ml	Specific activity (SA) U/mg	Recovery (%)	Purification fold
Crude	402.5	21,131.85	52.5	100	1
Extractive fermentation	94.2	18,670	198.1	88	3.77
Gel filtration chromatography	8.5	7217	849.05	34.1	16.1
Ion exchange chromatography	2.22	2603.2	1172	12.3	22.32

Relative uncertainty for the total protein, enzyme activity and specific activity are u_r_ (TP) = 0.2 mg/ml, u_r_ (EA) = 0.02 U/ml, u_r_ (SA) = 0.02 U/mg.

### Fibrin plate assay

**Figure 7:**
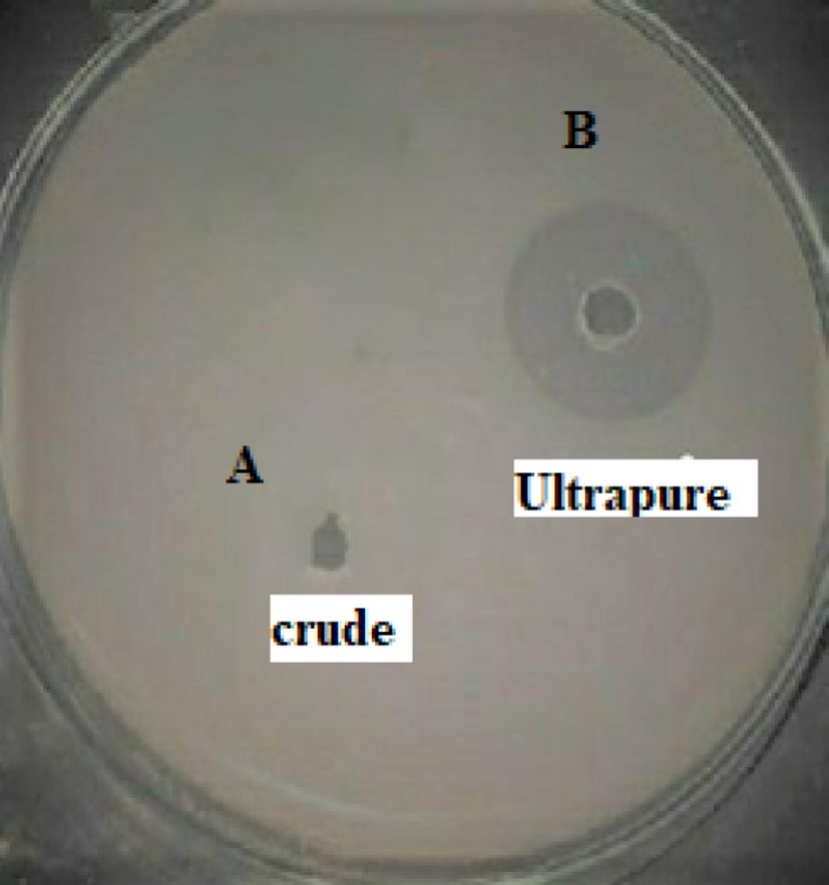
Fibrin plate assay for (**A**) crude fraction from micellar extractive fermentation and (**B**) Ultrapre fraction from anion exchange chromatography.

The extracted enzyme was screened for fibrinolytic activity by fibrin plate assay for the hydrolysis of fibrin. 10 µl of the crude, purified enzyme was added to the well and the zone of clearance was found to be 1.5 and 2.0 cm. From Fig. 7 it clearly shows that the ultrapure enzyme fraction was capable of degrading fibrin effectively.

## Conclusion

The present study proposed the concept of swappable pH-driven micellar two-phase extraction in the simultaneous production and purification of therapeutic enzyme at cloud point. Non-ionic surfactant deep eutectic solvents was investigated for the first time in extractive fermentation of therapeutic enzyme. In this study, SDESs, salt concentration and the pH played a key role on the partitioning of protease. The optimum conditions obtained was 35% SDES, 15% NA_2_SO_4_ and pH 6.3. The maximum yield of 88% fibrinolytic enzyme was achieved at top phase with purity fold of 22.24%. Therefore the study has established that the recovery of protease through micellar extractive fermentation based on non-ionic surfactant DES is a promising method for the purification of therapeutic enzyme from bacterial sources. The process integration of fermentation and simultaneous recovery method would be potential for the production of biomolecules. This method is also ecofriendly because of the usage of biodegradable solvents for the protease recovery. The SDESs recovery of 95% indicated that the proposed method is also economical for the extraction of protease.

## Supplementary Information

Supplementary Information.
