# Supplementary material for: Switchable deep eutectic solvent driven micellar extractive fermentation of ultrapure fibrin digesting enzyme from Bacillus subtilis
Source: Sci Rep. 2022 Jan 18;12:903. doi: 10.1038/s41598-022-04788-w (PMC8766521; doi:10.1038/s41598-022-04788-w)
Supplement: Supplementary file 1 — Supplementary Information. [file 41598_2022_4788_MOESM1_ESM.docx]

Fig S1: H^1^ NMR graph of SDES formed with Triton X 100 as HBD and Choline chloride as HBA (TX:CCL)

Fig S2: H^1^ NMR graph of SDES formed with Triton X 100 as HBD and Tetrabutyl ammonium chloride as HBA (TX:TBAC)

Fig S3: H^1^ NMR graph of SDES formed with Triton X 100 as HBD and Tetrabutyl ammonium bromide as HBA (TX:TBAB)

Fig S4: H^1^ NMR graph of SDES formed with Tween 80 as HBD and choline chloride as HBA (TW:CCL)

Fig S5: H^1^ NMR graph of SDES formed with Tween 80 as HBD and Tetra butyl ammonium chloride as HBA (TW:TBAC)

Fig S6: H^1^ NMR graph of SDES formed with Tween 80 as HBD and Tetra butyl ammonium bromide as HBA (TW:TBAC)
